# A neutralizing IL-11 antibody reduces vessel hyperplasia in a mouse carotid artery wire injury model

**DOI:** 10.1038/s41598-021-99880-y

**Published:** 2021-10-19

**Authors:** David Schumacher, Elisa A. Liehn, Pakhwan Nilcham, David Castaño Mayan, Chutima Rattanasopa, Kaviya Anand, Gustavo E. Crespo-Avilan, Sauri Hernandez-Resendiz, Roshni R. Singaraja, Stuart A. Cook, Derek J. Hausenloy

**Affiliations:** 1 Institute of Experimental Medicine and Systems Biology, University Hospital, RWTH Aachen University, Aachen, Germany; 2 Department of Anesthesiology, University Hospital, RWTH Aachen University, Aachen, Germany; 3 Department of Cardiology, Angiology and Intensive Medicine, University Hospital Aachen, Aachen, Germany; 4 Victor Babes National Institute of Pathology, Bucharest, Romania; 5 Department of Intensive Care and Intermediate Care, University Hospital, RWTH Aachen University, Aachen, Germany; 6 National Heart Research Institute Singapore, National Heart Centre, Singapore, 169609 Singapore; 7 Translational Laboratories in Genetic Medicine, Agency for Science, Research and Technology, Singapore, 138648 Singapore; 8 Yong Loo Lin School of Medicine, National University Singapore, Singapore, 169857 Singapore; 9 Cardiovascular Research Institute, National University Health System, Singapore, 119228 Singapore; 10 Cardiovascular and Metabolic Disorders Program, Duke-National University of Singapore Medical School, 8 College Road, Singapore, 169857 Singapore; 11 Department of Biochemistry, Medical Faculty, Justus Liebig-University, Giessen, Germany; 12 MRC LMS, London, W12 0NN UK; 13 The Hatter Cardiovascular Institute, University College London, London, WC1E 6BT UK; 14 Cardiovascular Research Center, College of Medical and Health Sciences, Asia University, Taichung, Taiwan

**Keywords:** Cardiovascular diseases, Vascular diseases, Atherosclerosis, Carotid artery disease, Restenosis

## Abstract

Vascular restenosis remains a major problem in patients with coronary artery disease (CAD) and peripheral artery disease (PAD). Neointimal hyperplasia, defined by post-procedure proliferation and migration of vascular smooth muscle cells (VSMCs) is a key underlying pathology. Here we investigated the role of Interleukin 11 (IL-11) in a mouse model of injury-related plaque development. *Apoe*^−/−^ mice were fed a hyperlipidaemic diet and subjected to carotid wire injury of the right carotid. Mice were injected with an anti-IL11 antibody (X203), IgG control antibody or buffer. We performed ultrasound analysis to assess vessel wall thickness and blood velocity. Using histology and immunofluorescence approaches, we determined the effects of IL-11 inhibition on VSMC and macrophages phenotypes and fibrosis. Treatment of mice with carotid wire injury using X203 significantly reduced post-endothelial injury vessel wall thickness, and injury-related plaque, when compared to control. Immunofluorescence staining of the injury-related plaque showed that X203 treatment did not reduce macrophage numbers, but reduced the number of VSMCs and lowered matrix metalloproteinase 2 (MMP2) levels and collagen content in comparison to control. X203 treatment was associated with a significant increase in smooth muscle protein 22α (SM22α) positive cells in injury-related plaque compared to control, suggesting preservation of the contractile VSMC phenotype. Interestingly, X203 also reduced the collagen content of uninjured carotid arteries as compared to IgG, showing an additional effect on hyperlipidemia-induced arterial remodeling in the absence of mechanical injury. Therapeutic inhibition of IL-11 reduced vessel wall thickness, attenuated neointimal hyperplasia, and has favorable effects on vascular remodeling following wire-induced endothelial injury. This suggests IL-11 inhibition as a potential novel therapeutic approach to reduce arterial stenosis following revascularization in CAD and PAD patients.

## Introduction

Despite advances in stent design and revascularization therapies, vascular restenosis remains a major problem in patients with coronary artery disease (CAD) and peripheral artery disease (PAD)^1–4^. In-stent restenosis can lead to severe complications such as cardiac ischemia and chronic limb threatening ischemia, and new therapeutic strategies are needed to prevent these complications. Vascular smooth muscle cells (VSMCs) switching from its contractile phenotype to a synthetic phenotype is a major contributor to neointimal hyperplasia, the key pathology underlying vascular restenosis^2,3^.

VSMCs are specialized cells found within the medial layer of the vasculature where their primary function is to regulate vessel tone and blood pressure. In response to vascular injury, VSMCs proliferate, migrate into the tunica intima and assume a synthetic phenotype which is an adaptive response but results in vessel wall thickening. The synthetic VSMC phenotype is characterized by secretion of extracellular matrix, leading to fibrosis and inflammation. The cellular transition to a synthetic phenotype is termed *phenotypic switching* and plays a key role in arterial restenosis, aortic remodelling, and the development of atherosclerosis^5–11^.

Two key factors associated with VSMC phenotypic switching and vascular pathologies such as atherosclerosis and arterial restenosis are transforming growth factor-beta (TGFβ) and angiotensin-II (ANGII)^12–14^. Fibroblast-to-myofibroblast differentiation and VSMC phenotypic switching share many similarities, including the secretion of extracellular matrix, cell proliferation and migration, and both transitions can be triggered by the same stimuli.

We have recently discovered that IL-11, a little studied cytokine of the IL-6 family, is important for fibroblast activation downstream of both TGFβ1 and ANGII as well as for VSMC phenotypic switching, in response to the same stimuli^15,16^. We hypothesized that IL-11 might play a role in vessel hyperplasia and investigated the effects of the neutralizing IL-11 antibody (X203) or an isotype control IgG antibody in a carotid wire-induced endothelial injury mouse model.

## Material and methods

All experiments and methods were performed in accordance with relevant guidelines and regulations. All animal experiments were performed in accordance with ARRIVE (Animal Research: Reporting of In Vivo Experiments) guidelines and approved by the Biomedical Sciences Institute Singapore Institutional Animal Care Committee at A*STAR (161165). All methods are reported in accordance with ARRIVE guidelines.

### Mouse husbandry

All experiments were approved by the Biomedical Sciences Institute Singapore Institutional Animal Care Committee at A*STAR (161165). Mice were maintained on a 12 h dark–light cycle, with ad libitum access to water and were fed with lipid-rich Western-Type Diet (D12079B, Research Diets, NJ), as indicated. Plasma for all experiments was isolated from blood withdrawn from the orbital sinus in EDTA coated capillary tubes. Plasma alanine aminotransferase (ALT), aspartate aminotransferase (AST), low-density lipoprotein (LDL-C), high-density lipoprotein (HDL-C), triglycerides (TG) and total cholesterol were measured using Cobas c111 (Roche Diagnostics, Switzerland).

### Carotid artery wire injury model of vascular restenosis

**Figure 1:**
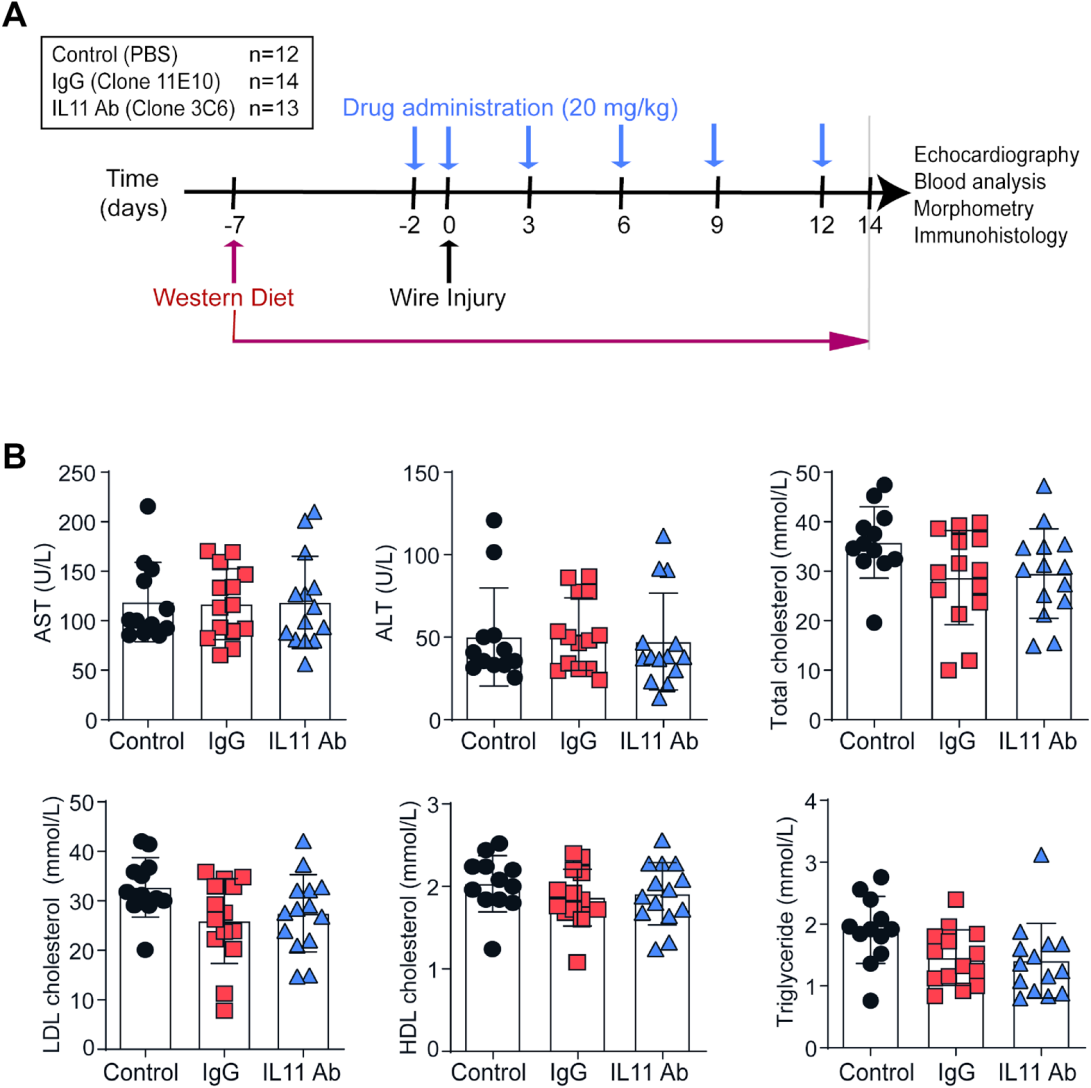
The effect of anti-IL-11 antibody (X203) treatment on plasma lipids. (**A**) A schematic of the experimental design. (**B**) Plasma concentration of AST, ALT, triglycerides, total cholesterol, LDL cholesterol and HDL cholesterol (N = 12–14/group, One-way ANOVA, Tukey’s multiple comparison test, Values: mean ± SD).

Male, 10 to 12 week *Apoe*^−/−^ mice (*C57BL/6J* background, Charles River Laboratory, Italy) were fed lipid-rich Western-Type Diet^17^ for a total of 3 weeks: 1 week before and 2 weeks after wire injury. Only male mice were used for this study to avoid the interference of estrogen effects on injury-related plaque with our target of interest IL-11. For the wire injury procedure, mice were anesthetized (100 mg/kg ketamine hydrochloride, 10 mg/kg xylazine i.p.) and subjected to endothelial denudation of the left common carotid artery using a 1 cm insertion of a flexible 0.36 mm guide wire through a transverse arteriotomy of the external carotid artery, as previously described^17^. Prior to surgery and for up to 2 days post-surgery, we performed analgesia with subcutaneous injection of Buprenorphine (0.05–0.1 mg/kg). Mice were randomly divided in three treatment groups: (1) PBS, (2) IgG, and (3) anti-IL-11 antibody treatment. Twenty mg/kg anti-IL-11 mouse monoclonal antibody (Clone 3C6; X203) or control IgG (Clone 11E10) were administered via intra-peritoneal injections^16^, twice per week beginning on the day of wire injury and continuing for 2 weeks (Fig. 1A).

X203 was generated in mice using a cDNA encoding amino acid 22–199 of human IL-11 cloned into expression plasmids (Aldevron Freiburg GmbH, Freiburg, Germany), as described previously^18^. Its efficacy has already been demonstrated in arterial remodeling^16^, myocardial infarction^15^, liver fibrosis^19^ and pulmonary fibrosis^18^.

### Ultrasound measurements of the carotid arteries and heart

Mice were anesthetized with 2% isoflurane and monitored to maintain heart rate above 500 beats/min during measurements. Measurements were performed in B-Mode and M-Mode. Velocities were recorded and measured in B-Mode (2D-realtime) using angle correction and vessel diameters (wall thickness) were recorded and analyzed in M-Mode using a 40 MHz transducer and a small-animal ultrasound imager (Vevo 3100, FUJIFILM Visualsonics, Toronto, Canada) as well as the VevoLab Software (FUJIFILM Visualsonics, Toronto, Canada).

### Histology and immunohistochemistry

Two weeks following wire injury, mice were anesthetized (100 mg/kg ketamine, 10 mg/kg xylazine, i.p.) and carotid arteries were excised, fixed in formalin and embedded in paraffin. The carotid arteries were then cut in 5 µm serial sections starting from the bifurcation until 500 µm, for all the collected sections. Verhoeff Van Gieson Elastic stain was performed as recommended by the manufacturer (ab150667, Abcam, Cambridge, UK) in 10 serial sections (50 µm apart, starting from the bifurcation) for the left side and 4 serial sections (50 µm apart, starting from the bifurcation for the right side). Injury-related plaque areas were determined for all sections using Diskus software (Hilgers, Königswinter, Germany), as previously described^17^. The average of all 10 sections for the left side (4 for right side) was considered as final restenosis area for each vessel.

For further measurements and to minimize variability of arterial layers after mechanical injury, we performed the measurements in whole vessel wall and have referred to it as injury-related plaque. Serial sections (3 sections per mouse, 100 µm apart) were stained to analyze the injury-related plaque and vessel for early differentiation of VSMCs (SM22α ab14106, Abcam, Cambridge, UK) and mature VSMCs (smooth muscle actin, M 0851 clone 1A4, DAKO, Germany), macrophages (Mac2, CL8942AP, Cedarlane, Germany) and MMP2 (ab110186, Abcam, Cambridge, UK). The sections were counterstained with DAPI for quantification of total cells. Positive-stained cells were counted in the injury-related plaque in each section and expressed as cells per injury-related plaque, percentage of all cells or percentage of positive area from the total injury-related plaque area. The results are represented as average of the measurements of all 3 slides.

Three serial sections, 100 µm apart, were stained with Gomori’s 1-step trichrome stain (ab150686, Abcam, Cambridge, UK). Blue-stained collagen content was analyzed with Cell P Software (Olympus, Hamburg, Germany) and expressed as a percentage of the injury-related plaque area. Final results were represented as average of the measurements of all 3 slides.

### Statistical analysis

Data are presented as mean ± SD. Statistical analysis were performed with Prism 6.1 software (GraphPad). For analyses between more than 2 groups we used 1-way ANOVA followed by Tukey’s multiple comparison test. *P* values of < 0.05 were considered significant.

## Results

### X203 treatment for 2 weeks did not affect blood lipids

To investigate the effect of IL-11 inhibition on neointimal hyperplasia, we performed wire injury in mice randomly assigned to receive either control IgG, the anti-IL-11 antibody X203, or no treatment (Fig. 1A). Since circulating lipids play an important role in arteriosclerosis and neointimal hyperplasia, we first assessed plasma lipids. With short term treatment, there were no differences in triglyceride, total cholesterol, LDL cholesterol or HDL cholesterol levels in mice across experimental groups (Fig. 1B). Furthermore, liver transaminases were similar in all treatment groups (Fig. 1B**)** suggesting that systemic anti-IL-11 antibody administration did not affect plasma lipids or liver function over the experimental time course.

### X203 treatment reduced post-wire injury neointimal hyperplasia

**Figure 2:**
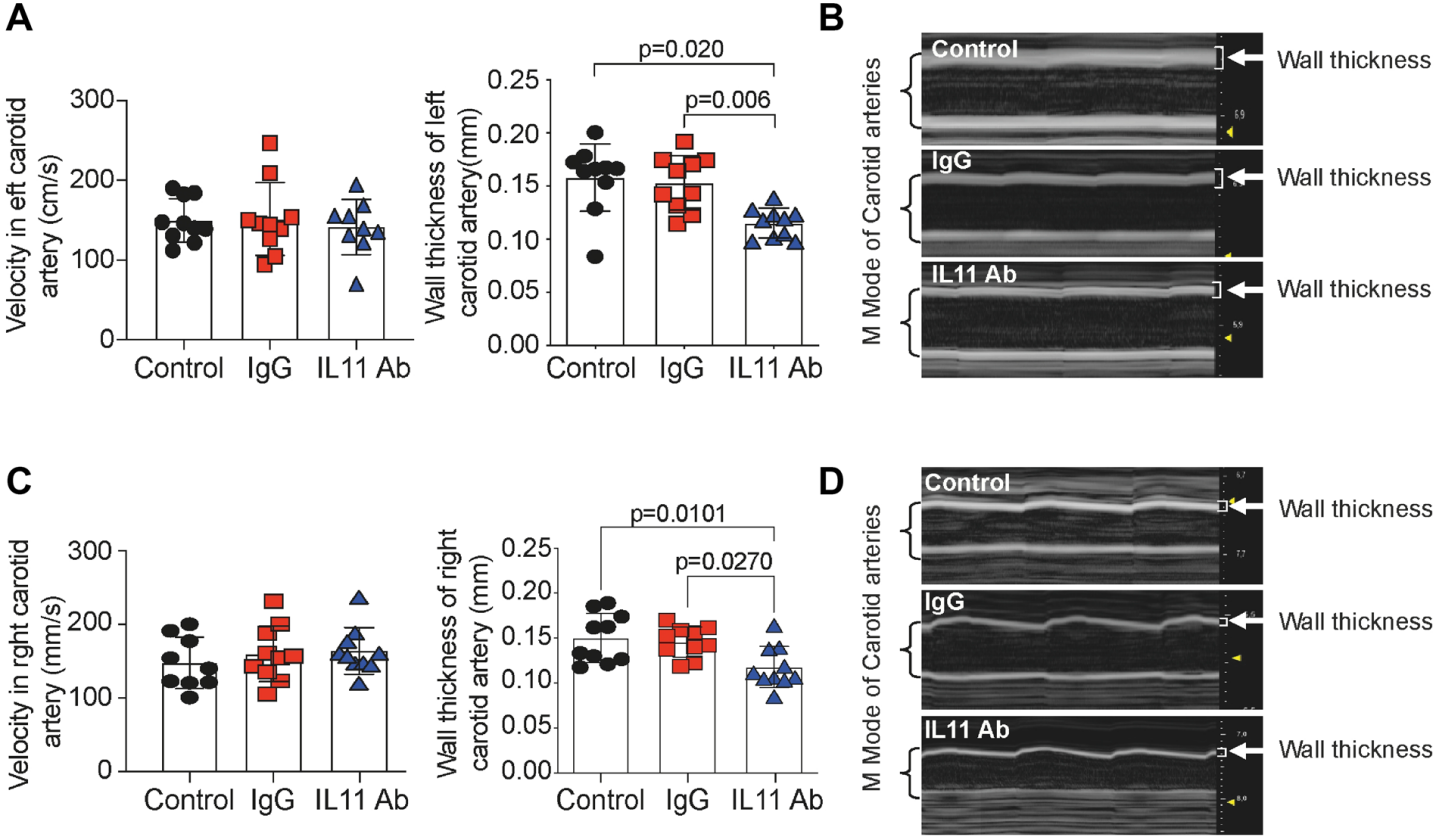
The effect of anti-IL-11 antibody (X203) treatment on vessel wall thickness. (**A**) Velocity and wall thickness of injured left carotid artery (N = 10/group, One-way ANOVA, Tukey’s multiple comparison test, Values ± SD). (**B**) Representative M-Mode images of injured left carotid artery. Brackets show carotid artery size, and arrows point out the measured wall thickness. (**C**) Velocity and wall thickness of uninjured right carotid artery (N = 10/group, One-way ANOVA, Tukey’s multiple comparison test, Values ± SD). (**D**) Representative M-Mode images of uninjured right carotid artery. Brackets show carotid artery size, and arrows point out the measured wall thickness.

To determine the effect of IL-11 inhibition on neointimal hyperplasia, we performed ultrasound analyses of injured carotid arteries. Wall thickness was significantly reduced in the X203 treated group compared to controls, whilst there were no differences in blood flow velocity (Fig. 2A,B). Interestingly, the ultrasound measurements of right-side, uninjured carotid artery show significant thinning after X203 treatment, while velocity showed no differences (Fig. 2C,D), demonstrating the effect of the X203 treatment on arterial remodeling. Original acquired ultrasound images are now presented in a supplementary figure (Suppl. Fig. 1).

**Figure 3:**
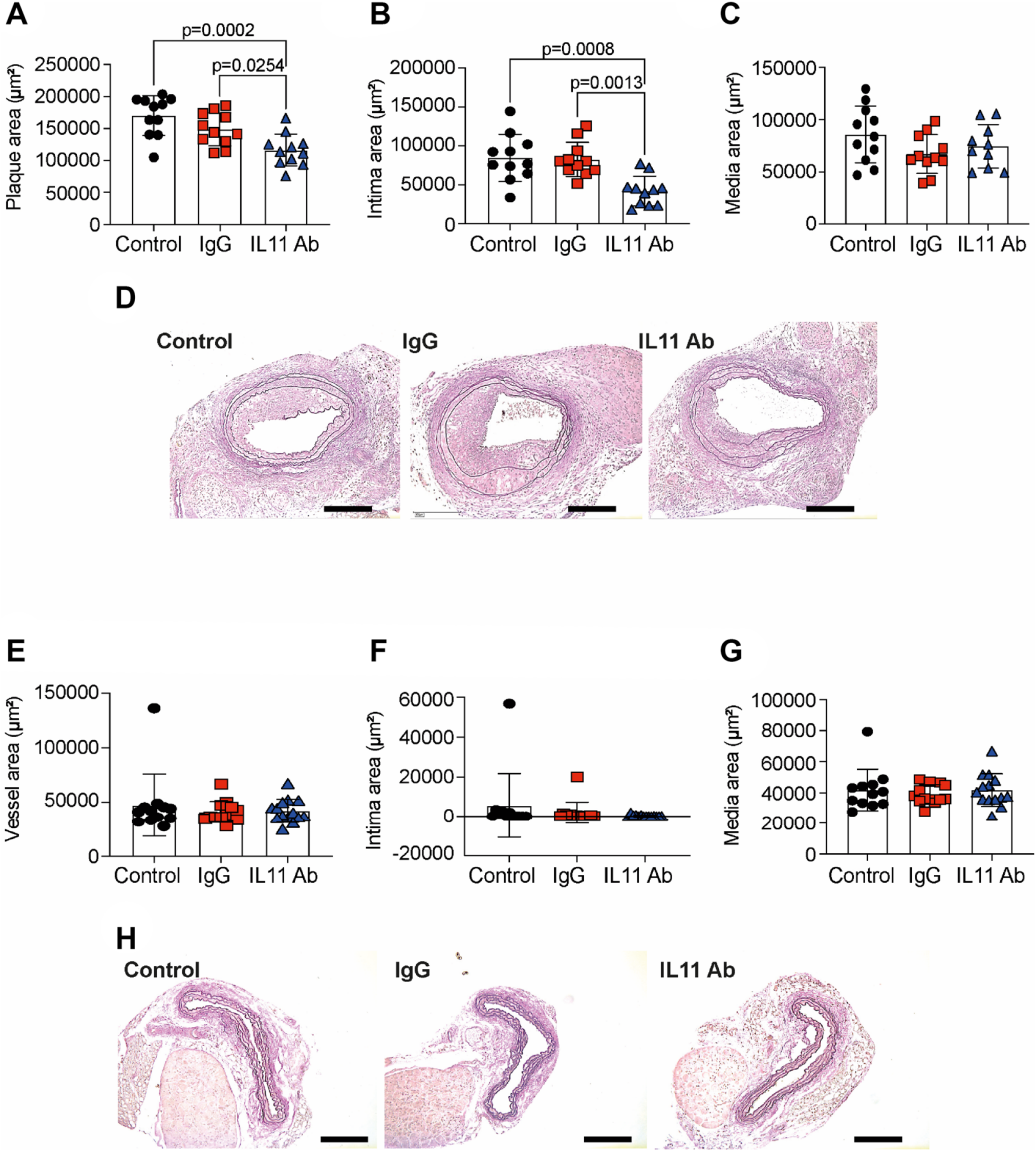
The effect of anti-IL-11 antibody (X203) treatment on neointimal hyperplasia. (**A**) Injury-related plaque area (N = 11/group, One-way ANOVA, Tukey’s multiple comparison test, Values ± SD). (**B**) Intima area (N = 11/group, One-way ANOVA, Tukey’s multiple comparison test, Values ± SD). (**C**) Media area (N = 11/group, One-way ANOVA, Tukey’s multiple comparison test, Values ± SD). (**D**) Representative images of Verhoeff Van Gieson Elastic stain (scale bar 200 μm). (**E**) Vessel area of control uninjured right carotid arteries (N = 12–14/group, One-way ANOVA, Tukey’s multiple comparison test, Values ± SD). (**F**) Intima area (N = 12–14/group, One-way ANOVA, Tukey’s multiple comparison test, Values ± SD). (**G**) Media area (N = 12–14/group, One-way ANOVA, Tukey’s multiple comparison test, Values ± SD). (**H**) Representative images of Verhoeff Van Gieson Elastic stain (scale bar 200 μm).

In addition, injury-related plaque (Fig. 3A), neointimal areas (Fig. 3B) were significantly reduced in X203-treated mice compared to controls. There were no differences in tunica media area between the treatment groups (Fig. 3C) Representative images are shown in Fig. 3D. Analyzing the right, uninjured carotid arteries, we found no differences in total vessel area (Fig. 3E), intima (Fig. 3F) or media (Fig. 3G). Representative images are shown in Fig. 3H. One out of 12 control carotid arteries (Suppl. Fig. 2A) and one out of 13 IgG-treated carotid arteries developed native atherosclerotic plaques, whereas none of the 14 X203-treated carotid arteries developed native atherosclerotic plaque.

### X203 treatment had no effect on post-endothelial injury macrophage injury-related plaque infiltration

**Figure 4:**
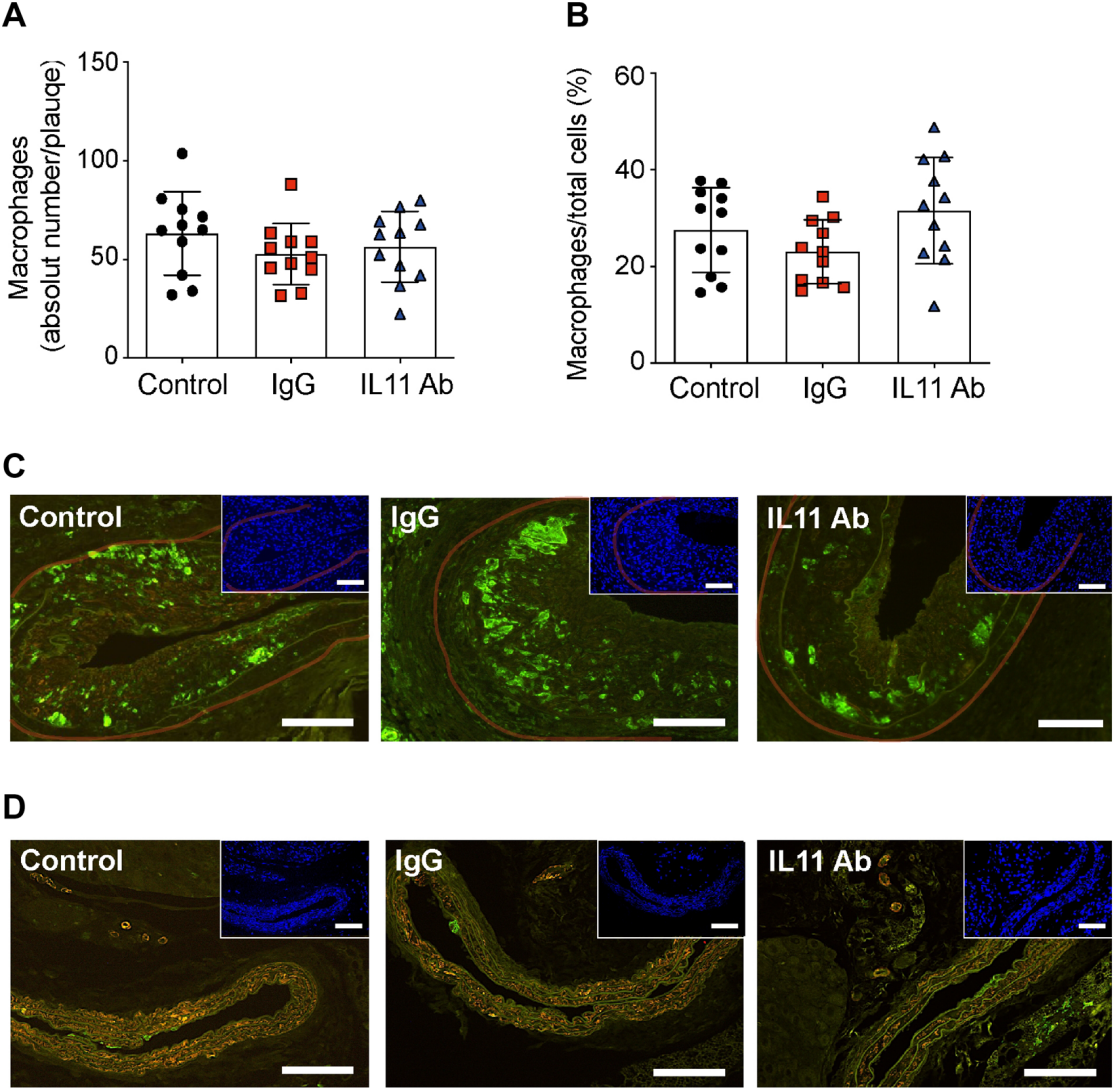
The effect of anti-IL-11 antibody (X203) treatment on macrophage infiltration. (**A**) Macrophages per injury-related plaque (N = 11/group, One-way ANOVA, Tukey’s multiple comparison test, Values ± SD). (**B**) Macrophage proportion of all cells (N = 11/group, One-way ANOVA, Tukey’s multiple comparison test, Values ± SD). (**C**) Representative images of Mac2 immunofluorescence staining and corresponding DAPI staining (insets) (scale bar 100 µm). Transparent red-lines were traced to delineate the injury-related plaque area used for quantifications. (**D**) Representative images of Mac2 staining in the right uninjured carotid arteries and corresponding DAPI staining (insets) (scale bar 100 µm).

Inflammation and macrophages play an important role in arteriosclerosis and vascular restenosis^20^. Therefore, we assessed the effect of IL-11 inhibition on the number of macrophages infiltrating the injury-related plaque area. Neither the proportion of macrophages nor the absolute number of macrophages were different across the treatment groups (Fig. 4A–C). The right carotid arteries showed no staining or isolated subendothelial staining for macrophage marker Mac2 (Fig. 4D), except the one carotid artery from the control groups presenting with native atherosclerotic plaque, which showed predominately macrophages infiltration (Suppl. Fig. 2B).

### X203 treatment reduced post-endothelial injury VSMC accumulation

**Figure 5:**
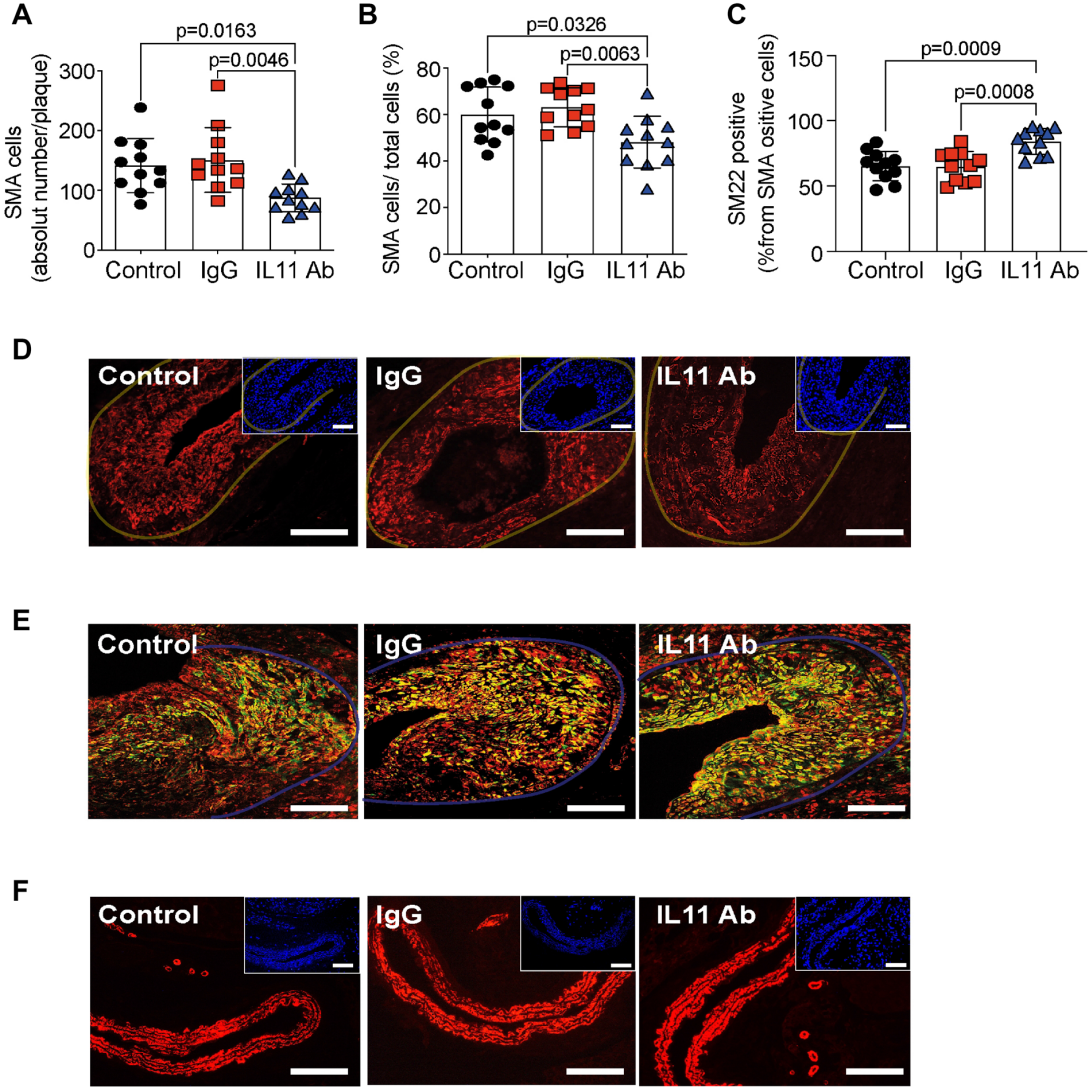
The effect of anti-IL-11 antibody (X203) treatment on injury-related plaque VSMC accumulation. (**A**) SMA^+^ VSMCs per injury-related plaque (N = 11/group, One-way ANOVA, Tukey’s multiple comparison test, Values ± SD). (**B**) SMA^+^ VSMCs percent of all cells (N = 11/group, One-way ANOVA, Tukey’s multiple comparison test, Values ± SD). (**C**) SM22α expression in VSMCs in the injury-related plaque (N = 11/group, One-way ANOVA, Tukey’s multiple comparison test, Values ± SD). (**D**) Representative images of SMA immunofluorescence staining (red) and corresponding DAPI staining (blue, insets) in left, injured carotid arteries (scale bar 100 µm). Transparent yellow-lines were traced to delineate the injury-related plaque area used for quantifications. (**E**) Representative images of SM22α (green) and SMA (red) immunofluorescence co-staining (yellow, scale bar 50 μm). Transparent blue-lines were traced to delineate the injury-related plaque area used for quantifications. (**F**) Representative images of SMA immunofluorescence staining (red) and corresponding DAPI staining (blue, insets) in right, uninjured carotid arteries (scale bar 100 µm).

Given the central role of VSMC switching to a synthetic phenotype characterized by proliferation and migration in post-endothelial injury neointimal hyperplasia, we investigated the effect of IL-11 inhibition on VSMC accumulation and phenotype. X203 treatment reduced the numbers of VSMCs in the injury-related plaque (Fig. 5A,B,D), and increased the numbers of VSMCs expressing SM22α, a contractile marker (Fig. 5C,E), when compared to controls, suggesting that most VSMCs in the injury-related plaques of the X203-treated mice were contractile in phenotype, suggesting atheroprotection. The right uninjured carotid arteries showed mainly vascular smooth muscle cells in the vessel wall (Fig. 5F).

### X203 treatment reduced post-endothelial injury injury-related plaque fibrosis

**Figure 6:**
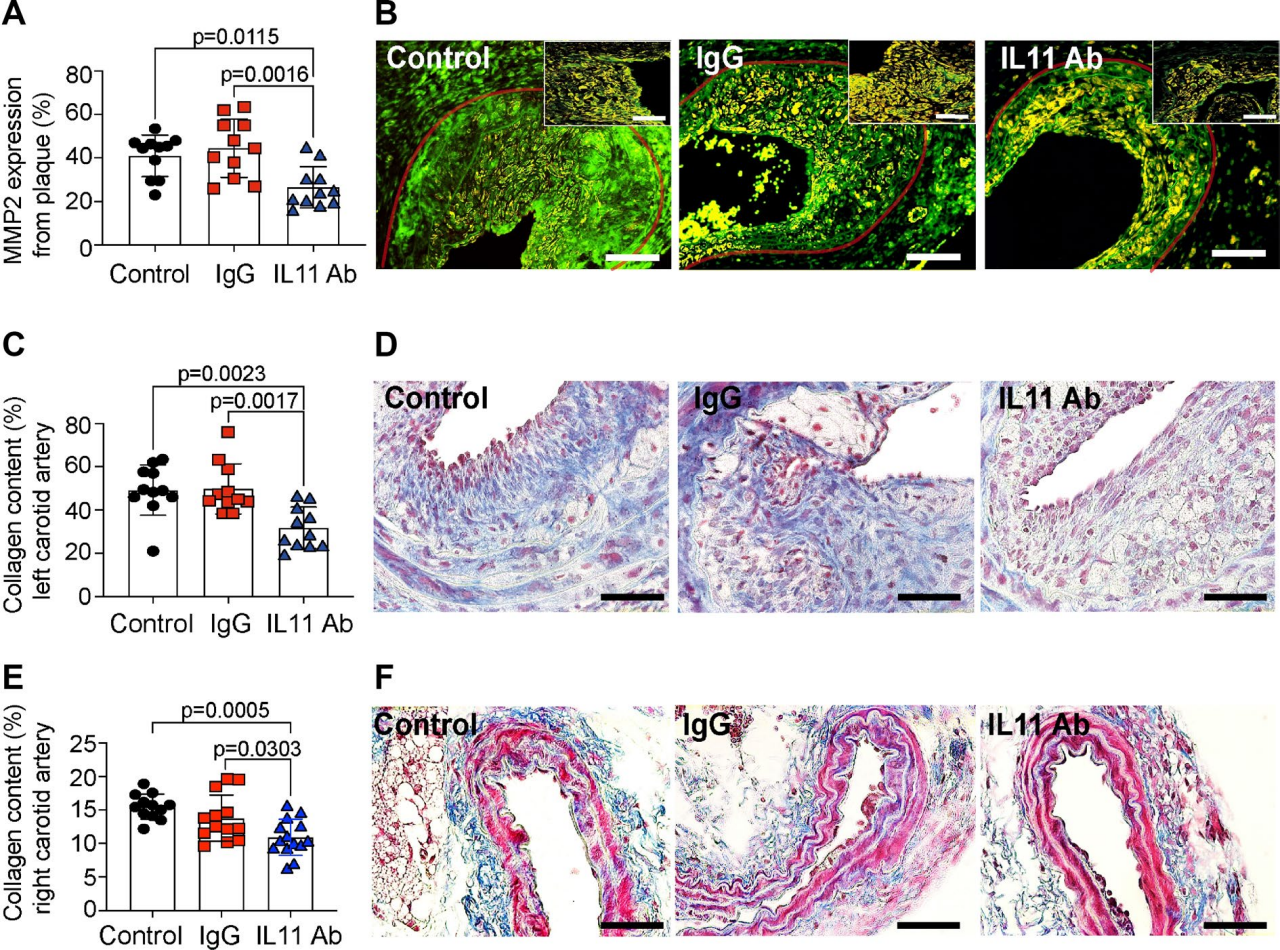
The effect of anti-IL-11 antibody (X203) treatment on post-endothelial injury injury-related plaque fibrosis. (**A**) MMP2 positive staining per injury-related plaque (N = 11/group, One-way ANOVA, Tukey’s multiple comparison test, Values ± SD). (**B**) Representative images of MMP2 immunofluorescence staining (green, scale bar 100 μm). Insets represent higher magnification of positive cells inside the restenosis plaque (scale bar 50 μm). Transparent red-lines were traced to delineate the injury-related plaque area used for quantifications. (**C**) Collagen content (blue) of the injury-related plaque (N = 11/group, One-way ANOVA, Tukey’s multiple comparison test Values ± SD). (**D**) Representative images of Gomori stain (collagen in blue, muscle in red, scale bar 50 μm). (**E**) Collagen content (blue) of right, uninjured carotid arteries (N = 12–14/group, One-way ANOVA, Tukey’s multiple comparison test Values ± SD). (**F**) Representative images of Gomori stain (collagen in blue, muscle in red, scale bar 50 μm).

Several recent studies have reported the pro-fibrotic properties of IL-11^15,16^. Given the critical role of VSMC switching to a synthetic phenotype in post-endothelial injury neointimal hyperplasia, which is characterized by the secretion of extracellular matrix, we investigated the effect of IL-11 inhibition on MMP2 expression and collagen content. X203 treatment decreased MMP2 expression (Fig. 6A,B) and reduced collagen content (Fig. 6C,D) in the injury-related plaque, compared to controls, again suggesting that inhibition of IL-11 is atheroprotective via reducing VSMC phenotype switching. Interestingly, the uninjured right carotid arteries showed a significant reduction of collagen content after X203 treatment (Fig. 6E,F), demonstrating the anti-fibrotic role of X203 treatment and protection against arterial remodeling. This may account for the right carotid arteries appearing thinner during the ultrasound measurements.

## Discussion

We demonstrate for the first time that inhibition of IL-11 reduces vessel wall thickness and neointimal hyperplasia in a carotid wire-induced endothelial injury mouse model. These findings were associated with beneficial effects on post-endothelial injury injury-related plaque remodelling as evidenced by decreased accumulation of VSMCs, increased proportion of contractile VSMCs, lower MMP2 expression, and reduced collagen content in the injury-related plaque. Interestingly, inhibition of IL-11 reduced the collagen content of uninjured carotid arteries as compared to either IgG or buffer control mice, showing an additional effect on hyperlipidemia-induced arterial remodeling in the absence of mechanical injury.

Autocrine IL-11 signaling is a key downstream effector of TGFβ1 and ANGII in different cell types involved in extracellular matrix (ECM) production^15,16,18,19^. In cardiac fibroblasts^15^, lung fibroblasts^18^ and hepatic stellate cells^19^, IL-11 is required for ERK-dependent myofibroblast activation. Interestingly, TGFβ1 induces IL-11 secretion from aortic and coronary artery VSMCs^21,22^. Recently, we showed a role of IL-11 in phenotypic switching of VSMCs and discovered the existence of an autocrine loop of IL-11 activity in VSMCs, which is required downstream of both TGFβ1 and ANGII for phenotypic switching to occur in the context of aortic modelling^16^. However, the role of IL-11 in phenotypic switching and function of VSMCs in the setting of neointimal hyperplasia following endothelial injury has not been investigated. It is known that X203 treatment has a positive effect on arterial remodeling in the context of hypertension^16^, on cardiac fibrosis and healing after myocardial infarction^15^, on liver fibrosis^19^ and in idiopathic pulmonary fibrosis^18^.

In this study, we showed that inhibiting IL-11 reduced neointimal proliferation and had favorable effects on injury-related plaque remodelling following wire-induced endothelial injury in the mouse carotid artery. As expected, carotid wire injury induced neointimal hyperplasia in control mice as evidenced by an increase in vessel wall thickness and tunica intima area, and treatment with IgG had no effect on these parameters. Treatment with the anti-IL-11 antibody X203 significantly reduced vessel wall thickness (with no effects on carotid artery velocity) and decreased total injury-related plaque area (with a reduction in tunica intima area but no effect on media area). The reduction in injury-related plaque area with X203 treatment was associated with decreased numbers of VSMCs, with an increased expression of SM22α, a marker for VSMCs with a preserved contractile phenotype^23^. These findings are consistent with our prior study showing that genetic or antibody-mediated inhibition of IL-11 attenuated VSMC phenotypic switching^16^. Several studies have identified macrophages to be important contributors to vascular restenosis^24,25^. However, we found no differences in macrophage injury-related plaque infiltration with X203 treatment, suggesting that the beneficial effects of inhibiting IL-11 on reducing neointimal hyperplasia were independent of macrophage accumulation into the injury-related plaque at this timepoint.

In our previous study we showed that IL-11 induces phenotypic switching of VSMCs to a synthetic phenotype characterized by secretion of collagen and extracellular matrix proteins, including MMP2^16^, which is involved with phenotype switching and migration^26,27^. Furthermore, expression of the VSMC contractile marker SMA22α was also decreased in response to IL-11 antibody treatment in the same study^16^. Consistent with a pathological role for IL-11 on VSMC function, we show here that treatment with X203 decreased injury-related plaque MMP2 levels and increased injury-related plaque SMA22α levels, suggesting a favorable effect of IL-11 inhibition on vascular remodelling following wire-induced endothelial injury.

We highlight that while IL-11 was discovered three decades ago, there is very little known of its effects in the vasculature. Limited earlier studies have suggested IL-11 as anti-inflammatory, anti-fibrotic and pro-regenerative^28^ and in the vasculature, IL-11 has been thought to inhibit VSMC proliferation and plaque formation^29^, the opposite of what we demonstrate here. One reason for the general misunderstanding of IL-11 function relates to the repeated use of recombinant human IL-11 in mouse models of disease. Paradoxically, it was recently shown that rhIL-11 is a competitive inhibitor of mouse IL-11 in mouse cells and thus much of the earlier literature may need to be reconsidered^30^.

In conclusion, we show for the first time that inhibition of IL-11 reduced neointimal hyperplasia following endothelial injury and had favorable effects on vascular remodelling. These findings position the IL-11 antibody (X203) as a novel therapeutic strategy for preventing post-angioplasty/stent restenosis and improving outcomes in CAD and PAD patients undergoing revascularization.

## Supplementary Information

Supplementary Information.

## Abbreviations

IL-11: Interleukin-11
VSMC: Vascular smooth muscle cells
TGF-β: Transforming growth factor-beta
ANGII: Angiotensin-II
SMA: Smooth muscle actin
MMP2: Matrix metalloproteinase 2
SM22 α: Smooth muscle protein 22α
