# Supplementary material for: A neutralizing IL-11 antibody reduces vessel hyperplasia in a mouse carotid artery wire injury model
Source: Sci Rep. 2021 Oct 19;11:20674. doi: 10.1038/s41598-021-99880-y (PMC8526715; doi:10.1038/s41598-021-99880-y)
Supplement: Supplementary file 1 — Supplementary Information. [file 41598_2021_99880_MOESM1_ESM.docx]

**Supplementary Figure 1**

**
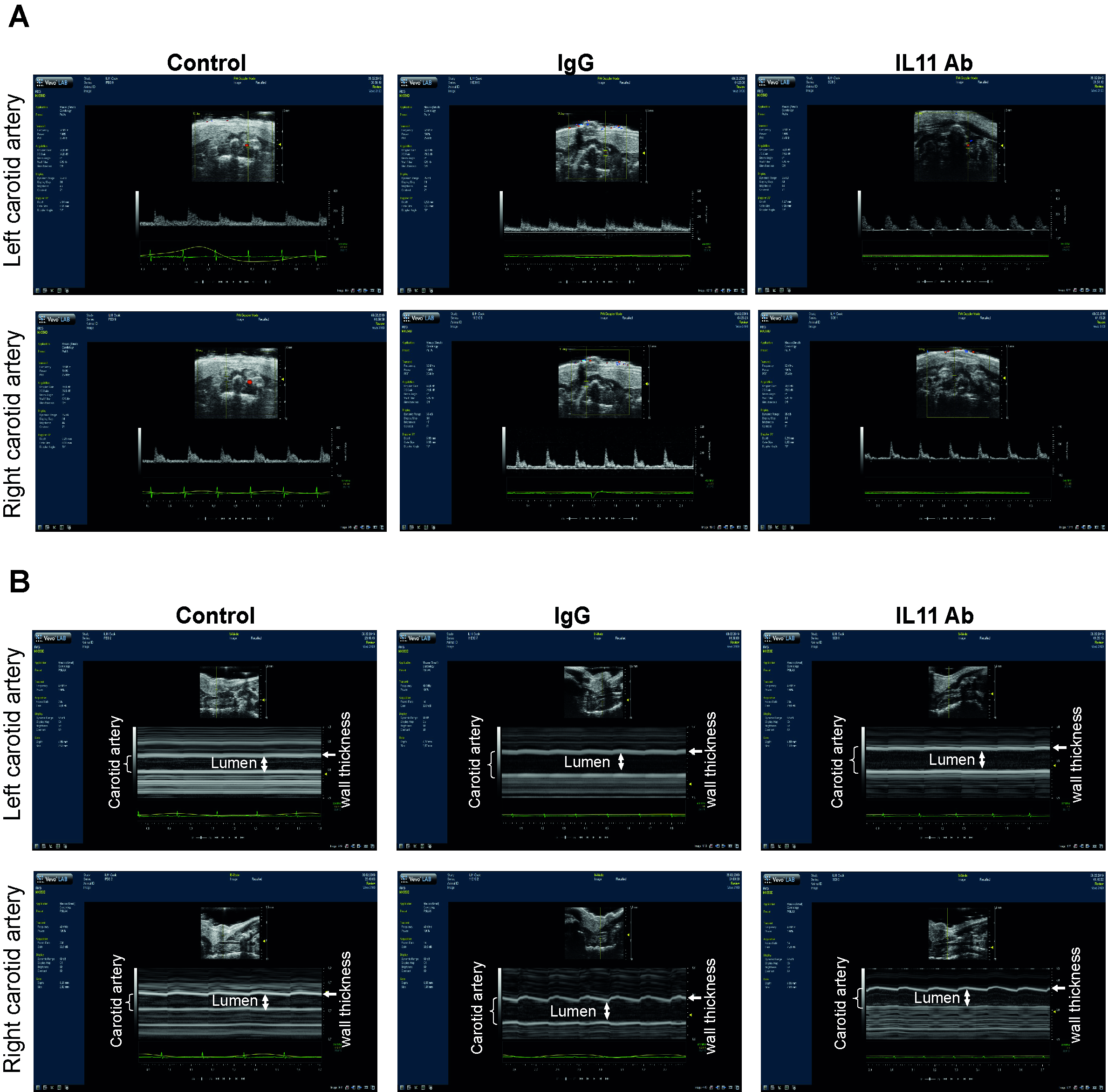
**

*Original acquired ultrasound images using 40 MHz transducer and small-animal ultrasound imager (Vevo 3100)*. (A) Velocity was recoded in B-Mode (2D-realtime, lower panel) using angle correction in both, injured and uninjured carotid arteries. (B) Measurements of vessel wall thickness were performed in M-Mode. Brackets show the carotid artery and arrows point to the measured vessel wall.

**Supplementary Figure 2**

**
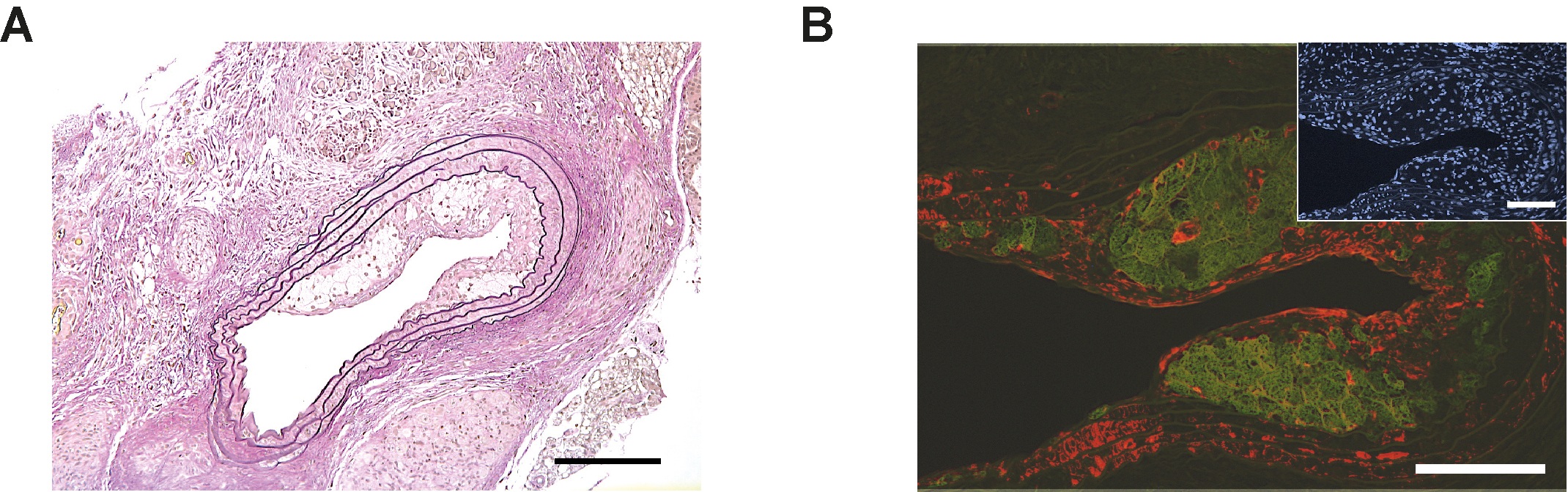
**

*Native atherosclerotic plaque in an untreated control right carotid artery.* (A) Verhoeff Van Gieson Elastic stain (scale bar 200 μm). (B) Images of Mac2 immunofluorescence staining (in green) and smooth muscle cells (in red) and corresponding DAPI staining (inset) (scale bar 100 µm).
